# Nanoparticle Properties and Synthesis Effects on Surface-Enhanced Raman Scattering Enhancement Factor: An Introduction

**DOI:** 10.1155/2015/124582

**Published:** 2015-03-25

**Authors:** Nathan D. Israelsen, Cynthia Hanson, Elizabeth Vargis

**Affiliations:** Department of Biological Engineering, Utah State University, 4105 Old Main Hill, Logan, UT 84322, USA

## Abstract

Raman spectroscopy has enabled researchers to map the specific chemical makeup of surfaces, solutions, and even cells. However, the inherent insensitivity of the technique makes it difficult to use and statistically complicated. When Raman active molecules are near gold or silver nanoparticles, the Raman intensity is significantly amplified. This phenomenon is referred to as surface-enhanced Raman spectroscopy (SERS). The extent of SERS enhancement is due to a variety of factors such as nanoparticle size, shape, material, and configuration. The choice of Raman reporters and protective coatings will also influence SERS enhancement. This review provides an introduction to how these factors influence signal enhancement and how to optimize them during synthesis of SERS nanoparticles.

## 1. Introduction

Raman spectroscopy is a vibrational spectroscopy technique that has great promise for the identification of solids, liquids, and gases. Similar to infrared (IR) spectroscopy, Raman spectroscopy is a technique used to study molecular vibrations. One advantage that Raman spectroscopy has over traditional IR spectroscopy is that it avoids elaborate sample preparation. Despite this advantage, Raman spectroscopy was not initially as popular as IR spectroscopy due to difficulty in detecting Raman scattering. Statistically, for every 1–10 million photons bombarding a sample, only one will result in Raman scattering [1, 2]. Thanks to technological advances such as mercury lamps, lasers, spectrometers, and charge-coupled devices (CCDs), Raman has gained popularity as a means of chemical identification [3]. Raman continued to gain traction with the aid of signal enhancement methods. In 1974, three scientists from Southampton University (Fleischmann, Hendra, and McQuillan) observed that the Raman spectrum of pyridine on a roughened silver electrode showed unusually large inelastic light scattering [4]. The phenomenon, later termed surface-enhanced Raman spectroscopy (SERS), can enhance the inelastic light scattering events by a factor of 10^6^ or more [5]. This enhancement allows researchers to develop extremely sensitive methods for quantification and identification of small molecules and molecular biomarkers.

Two mechanisms are behind the increased inelastic light scattering seen in SERS, electromagnetic and chemical enhancement. The electromagnetic enhancement is the dominant effect and is due to localized surface plasmon resonance (LSPR). LSPR is an optically excited electron wave resonance state on the surface of a nanostructure, which can increase both elastic (Rayleigh) and inelastic (Raman) light scattering from the sample [6]. The chemical enhancement mechanism is caused by molecular charge-transfer interactions between the molecule and the metallic surface [7, 8]. Together, these mechanisms of enhancement increase the Raman intensity to a point where SERS can be used for applications which require greater molecular sensitivity.

There are two SERS methodologies, intrinsic and extrinsic SERS. Intrinsic SERS involves the direct measurement of the target molecule. In this process, a unique spectrum of the molecular vibration is acquired, which is referred to as a Raman signature. Intrinsic SERS is advantageous in understanding structural information about the target molecule and providing qualitative differences between similar samples. Intrinsic SERS has been used for many biological applications such as detecting small molecules like glucose [9, 10], antioxidants [11], and amino acids [12]. It has also been used to detect macromolecules such as proteins [13] and DNA [14]. Drawbacks to intrinsic SERS include insensitivities of the molecule to Raman shifts and difficulty in detecting low concentrations. In cases of very dilute samples or where the sample is fairly Raman insensitive, extrinsic SERS provides an alternative mode of detection.

**Figure 1:**
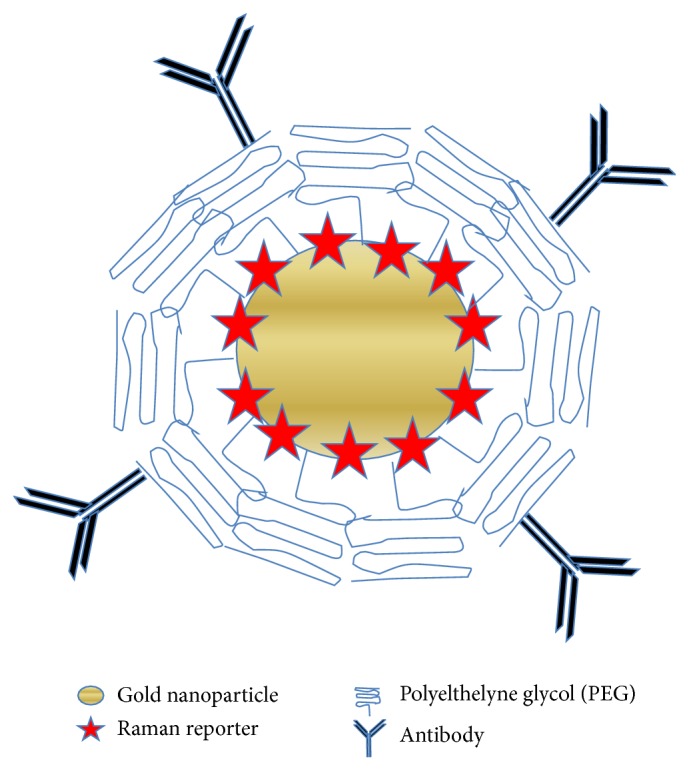
An illustration of an extrinsic SERS nanoparticle for targeting of a specific antigen.

Extrinsic SERS is an indirect method of measuring target molecules because the acquired spectrum is of a Raman reporter rather than the target itself. A Raman reporter is a molecule that has inherently strong Raman active modes. An illustration of a nanoparticle used for extrinsic SERS is provided in Figure 1, where a Raman reporter molecule is bound to the metal surface and encased in a protective layer. This protective layer prevents nanoparticle aggregation and reporter leaching by steric hindrance and charge neutralization. The nanoparticle is then functionalized with an antibody or other ligand to target specific molecular sites. Nanoparticles for extrinsic SERS can be applied in a variety of situations where it is difficult to take intrinsic SERS measurements. For example, nanoparticles used for extrinsic SERS can target specific cell biomarkers [15] and cancer cells [16], while intrinsic SERS applications do not have targeting capabilities. In addition, nanoparticles for extrinsic SERS are synthesized to prevent aggregation in a variety of environments [17]. This trait accommodates* in vivo* measurements. For example, extrinsic SERS has been used to measure* in vivo* liver function [18]. In contrast, nanoparticles used in intrinsic SERS applications are susceptible to aggregation, preventing analysis in certain environments.

Regardless of the SERS method employed, the SERS enhancement factor (EF) is calculated in a variety of ways [19]. However, the simplest and most used definition relies on an estimate of the number of adsorbed reporter molecules and is calculated as follows:

(1) $$\begin{array}{c} \text{EF}=\frac{I_{\text{SERS}\;}/N_{\text{SERS}}}{I_{\text{Normal}}/N_{\text{Normal}}}, \end{array}$$

where *I*_SERS_ is the SERS intensity, *N*_SERS_ is the number of molecules adsorbed to the metal surface in the SERS excitation volume, *I*_Normal_ is the non-SERS intensity of the solution, and *N*_Normal_ is the number of molecules in the non-SERS excitation volume. There has been some debate about this formula as it depends on several experimental conditions such as excitation wavelength, molecular species being evaluated, and molecular adsorption properties. Due to variations in these experimental conditions, it is difficult to make an accurate comparison of SERS EFs [19]. EFs are also difficult to compare as there are different types of EF measurements, the maximum EF and the average EF. The maximum EF, although larger, is seldom used in practice because it is difficult to locate SERS hot spots (locations of maximum SERS enhancement). The average SERS EF is the average enhancement for the scattering volume and is more repeatable. Maximum SERS EFs are typically on the order of 10^10^ while average SERS EFs are typically between 10^4^ and 10^6^ [19].

The lack of a rigorous definition of SERS EF and calculation errors has resulted in SERS EFs which have been reported as high as 10^14^ [5]. For example, in a study on single molecule detection of crystal violet, the SERS intensity of crystal violet is compared to the non-SERS intensity of methanol. This comparison resulted in a high SERS EF which is inaccurate because the author failed to account for the non-SERS intensity of crystal violet [19]. Other sources of error include failing to account for the following parameters: molecule orientation and surface selection rules [20], differentiation between average and maximum EF [21], and photobleaching [22]. In addition, errors can result from inaccurate measurements of metal bound analyte concentration due to shape irregularity and surface roughness [23]. A detailed discussion and rigorous definition of EF has been presented by Le Ru et al., and further details will not be discussed here [19].

The SERS enhancement effect has been observed with alkaline metals [24], various transition and noble metals [24, 25], metals oxides [25] and even semiconductor materials like silicone and graphene [25, 26]. The SERS enhancement of semiconductors is primarily due to chemical mechanisms and is weaker than the enhancement observed with noble metals [25]. Despite the lower enhancement, semiconductors have potential applications in the development of photodetectors and biosensors [27].

Typically, SERS is observed with coinage metals such as gold, silver, and copper due to the large SERS enhancement they produce. Of these metals, silver provides the highest enhancement factor due to its absorption properties. Optical absorption in metals is increased by interband transitions. The interband transition of silver is found in the ultraviolet range. As a result, there will be less absorption in the visible or near-IR Raman wavelengths resulting in large SERS intensities. In contrast, gold and copper have interband transitions in the visible wavelength range which results in a decrease in the maximum SERS intensity [28, 29]. Despite the greater enhancement capability of silver, gold is often used as it is more stable, is biocompatible, and has an easier surface chemistry than silver [30]. The decision to use gold or silver will depend on several factors such as application, excitation wavelength, LSPR wavelength, and experimental setup.

It has been reported that SERS enhancement is dependent on the distance between the SERS surface and the molecule of interest [31]. Although the target molecule does not need to be touching the surface for SERS enhancement to occur, literature suggests that it needs to be within 1–30 nm for reasonable SERS enhancement [32–34]. The actual distance for SERS enhancement is highly dependent on the nanoparticle surface. Calculations for metallic spherical nanoparticles have predicted that the SERS intensity will decay with increasing distance, *r*, between the target molecule and particle surface. The distance for electromagnetic enhancement is proportional to *r* ^−12^. Due to the addition of surface molecules, the decay relationship increases slightly and is proportional to *r* ^−10^ [31, 33].

To effectively optimize nanoparticle EF, the LSPR peak should be taken into account. Initially, it was proposed that the largest amount of Raman scattering should be observed when the particle was excited at its LSPR peak [35]. Experimental studies have found that the optimal excitation wavelength will be slightly blue-shifted from the nanoparticle LSPR peak [36]. One approach to estimating the optimal excitation wavelength for SERS enhancement is to design nanoparticles with an LSPR peak (*λ*_LSPR_) between the excitation wavelength (*λ*_0_) and the Raman wavelength of the interest (*λ*_*R*_). This can be expressed as *λ*_LSPR_ = (*λ*_0_ + *λ*_*R*_)/2 [37]. This approach has been validated by a number of independent researchers and appears to apply to many nanostructures investigated [37–39]. However, there are deviations from this rule due to the excitation wavelength used as well as the shape of the nanoparticle [40]. As a general guideline, SERS enhancement has been observed for excitation wavelengths from 600 nm to 1200 nm for gold and from 400 nm to 1000 nm for silver [41].

To synthesize SERS nanoparticles with an optimal EF for intrinsic applications, it is requisite to understand how a nanoparticle's material, size, shape, and configuration influence EFs. Synthesis of nanoparticles for extrinsic SERS applications requires additional understanding of how Raman reporters and protective layers influence EF. This paper provides an introduction to how these factors influence EF (Sections 2 and 3) and how to control them during synthesis (Sections 4 and 5).

## 2. Enhancement Dependency on Nanoparticle Properties for Intrinsic SERS

### 2.1. Enhancement Dependency on Nanoparticle Size

**Figure 2:**
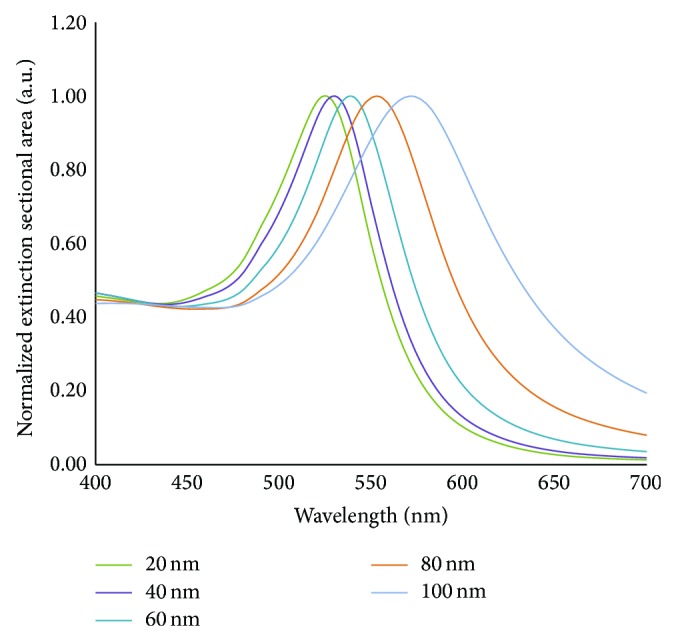
Normalized extinction cross-sectional area for spherical gold nanoparticles of diameters ranging from 20–100 nm. Data was obtained from a Mie theory simulation available online [42].

Enhancement and LSPR dependency on size is demonstrated in Figure 2, which shows the theoretical (Mie theory) extinction cross section for gold spheres ranging from 20 to 100 nms in diameter. The Mie theory is the solution to the Maxwell equations for how light interacts with a spherical particle. The theory predicts that the smallest spheres have a maximum LSPR at shorter wavelengths. Details concerning the Mie theory can be found in literature and will not be covered here [43, 44]. The relationship between LSPR wavelength and particle size has also been demonstrated experimentally [45–47].

The effect of nanoparticle size on SERS enhancement has been studied by several researchers [48–52]. Previous studies show that spherical gold particles with a 50 nm diameter produced the maximum SERS EF [48, 51]. Others have reported the optimal size particles for SERS enhancement to be in a range from 30 to 100 nm [50, 52]. Regardless of the exact size for optimal enhancement, there is an effective SERS range. When particles are too small, the effective conductivity and light scattering properties, which are needed for SERS enhancement, diminish [50]. As particles increase in size, the SERS effect increases as it depends on the number of electrons available [53]. When the particle size approaches the scale of the excitation wavelength, the particles become preferentially excited in nonradiative modes, leading to a diminished SERS effect [50].

### 2.2. Enhancement Dependency on Shape

Variations in particle shape can expand the LSPR range. For example, gold nanorods with aspect ratios from 1 to 19 have a LSPR peak range from 508 nm to 2135 nm [54]. Controlling particle shape and size allows researchers to accurately tune nanoparticles for a specific LSPR peak and optimize EF. Shape also influences SERS enhancement due to locations of high curvature such as sharp corners or tips. These locations produce unusually large electromagnetic enhancement. This enhancement is referred to as the lightning rod effect because similar to a pointed lightning rod, the electric field induced at the tip will be much stronger than other areas on the surface [55]. The influence of the lightning rod effect can be observed near high curvature points on many different shapes [56, 57]. The lighting rod effect allows nanostructures to act as an optical antenna providing an enhanced electromagnetic field [58–60]. More details about the lighting rod effect can be found in literature [61, 62].

### 2.3. Enhancement Dependency on Material and Configuration

SERS core-shell configurations can be made by coating a nanoparticle with gold or silver. Some common core materials include organic polymers [63–65], silica [66–68], iron oxide (Fe_3_O_4_) [69, 70], or other metals [71–73]. In core shell configurations, the materials used [73], the overall particle size [74], the shell thickness [75], and the core/shell ratio [74] will influence SERS EF due to shifts in the LSPR peak. For the purpose of this paper, only the influence of core-shell materials and shell thickness on SERS enhancement factor will be covered.

When two metals with different dielectric constants are placed next to each other in a core shell configuration, a LSPR shift will occur. By using the Mie theory, researchers have predicted that increasing the shell thickness of a nanoparticle will shift the LSPR peak to shorter wavelengths [75, 76]. This shift has been confirmed experimentally in a number of cases [75–77]. For example, Oldenburg et al. [75] observed a blue shift in the extinction peak as the gold shell thickness increased over a silica core once the shell was fully formed. The same trend can be seen in nanorods with a core-shell configuration. Ma et al. [76] observed how the LSPR peak shifted to lower wavelengths as the shell thickness increased for silver coated gold nanorods. Not only was there a blue shift, but the intensity also increased according to shell thickness. In the case of silver-gold core-shell arrangements, there will be a red shift as gold has a plasmon resonance peak at a longer wavelength than silver. As the gold shell thickness increases, the absorbance peak also diminishes [78, 79]. In addition to these changes, silver-gold or gold-silver core-shell arrangements create two plasmon resonance peaks [76, 78, 80] corresponding to silver and gold. Changes in LSPR wavelengths can be modified using core-shell nanoparticles by controlling composition materials and shell thickness among other parameters. The LSPR can be tuned to a position to create a maximum enhancement factor by proper placement in relationship to the excitation and emission wavelengths [40].

Another approach to SERS enhancement is due to SERS hot spots. They are locations between particle aggregates which amplify the electromagnetic field near the particle. Researchers have often used nanoparticle junctions or aggregates to create repeatable hot spot locations for single molecule SERS measurements [81–83]. By carefully controlling the placement of the reporter molecule within the particle junction, a maximum SERS EF is observed because hot spot locations are known in advance [84].

## 3. Enhancement Dependency on Nanoparticle Properties for Extrinsic SERS

### 3.1. Enhancement Dependency on Raman Reporter Properties

Fluorescent dyes and other chromophores are often used as Raman reporters because they have large Raman scattering cross sections, are photostable, and have resonance Raman capabilities. In resonance Raman scattering, the laser excitation wavelength is matched to the reporter molecule's absorption maximum and an approximate 10^3^–10^6^ increase in Raman scattering occurs [95]. By carefully matching the excitation wavelength and nanoparticle composition, surface-enhanced resonance Raman scattering (SERRS) can be observed. SERRS is especially suited for extrinsic measurements if the reporter molecule and excitation wavelengths are known before analysis and can be selected to provide the highest enhancement possible.  Table 1 shows molecules that have been used for SERRS studies.

Other characteristics which are important when selecting a Raman reporter molecule are its polarizability, photostability, and binding affinity. Polarizability is the ability of an external electromagnetic field to produce a change in the electron distribution in the molecule. Polarizable molecules have bond configurations which allow an excitation of the vibrational modes of the molecule in response to incident light. A Raman active molecule has a net change in its polarizability when excited by light which causes a shift in the wavelength of the scattered light [96]. Raman reporters should be highly Raman active.

The photostability of a Raman reporter molecule plays an important role in the SERS EF and in determining the maximum laser power to use. Photobleaching effects can account for SERS EF differences on the order of 10^2^–10^3^, which is comparable to the EF contribution due to chemical enhancement [97]. Photobleaching effects are related not only to the power of the incident light but also to the actual electromagnetic enhancement experienced by the reporter. As a result, even non-resonant molecules may experience photobleaching [98]. Due to the photobleaching effect, SERS nanoparticles experience a laser power dependent SERS EF. Lower laser power will reduce photobleaching effects and result in more consistent SERS measurements [97]. The use of near IR or IR lasers for excitation will also reduce the photobleaching effect and is recommended for photosensitive reporters [99].

Molecules with strong binding affinities to a gold or silver surface give stronger enhancement due to the chemical enhancement mechanism [91]. High binding affinity molecules that are frequently used are thiol or amine containing molecules because they will easily bind to the gold surface through the gold thiolate bond. Also, positively charged molecules such as crystal violet can associate with the negatively charged nanoparticle surface and are frequently used as Raman reporters [90]. Qian et al. showed how strong binding affinity affects the SERS enhancement factor when using two very similar Raman reporters: malachite green and malachite green isothiocyanate (MGITC) [91]. Although both molecules have similar Raman scattering cross sections and vibrational modes, MGITC showed an approximate 200-fold higher SERS EF under identical measurement conditions. They attributed this EF increase to stable anchoring of the MGITC molecule (by the isothiocyanate group) which enabled charge transfer and chemical enhancement to occur [91].

### 3.2. Enhancement Dependency on Protective Coating

The SERS protective coating can influence the enhancement effect due to its laser transparency and binding affinity. Coating materials must allow laser light to penetrate the coating and interact with the Raman reporters. Many materials have been used and include polymers [100], silica [101], or proteins [102]. Some coating materials can have an adverse effect on SERS response. For example, Huang et al. [103] observed a 60% decrease in the SERS signal due to the silica coating. Often, (3-mercaptopropyl)trimethoxysilane (MPTMS) is used as a silane functionalizing group because of its ability to bind to the gold surface through a thiolate bond. When the reporter and MPTMS are added simultaneously, the sulfur group binds preferentially, decreasing reporter density and the resulting SERS intensity [104]. This effect highlights the importance of controlling the reporter density on the particle surface as this will have an impact on the SERS enhancement.

## 4. Controlling Nanoparticle Geometry

The two main approaches for nanoparticle synthesis are the top-down and bottom-up approaches. The top-down approach starts with larger particles and disperses them to smaller particles. The bottom-up approach starts with smaller particles which build upon each other to form nanoparticles [105]. Top-down approaches typically produce particles in solution that are not as stable or reproducible as those produced from bottom-up approaches [105]. Therefore, this section will focus on bottom-up methods. Some examples of bottom-up approaches include chemical reduction [106–109], sonochemical synthesis [63, 110, 111], photochemical reduction [112], and radiolytic reduction [113]. As this is an introduction to optimal SERS nanoparticle synthesis, the most common chemical reduction methods will be covered.

Chemical reduction methods for synthesis of gold or silver nanoparticles incorporate the use of capping and reducing agents. A capping agent binds to the surface of the nanoparticle and prevents aggregation by repulsive or steric forces. It can also be used to functionalize nanoparticles in extrinsic SERS applications by providing a link to bind the nanoparticle to reporters and antibodies. Some examples include sodium citrate [106], dodecanethiol [107], thiol polyethylene-glycol (PEG) [114], cetrimonium bromide (CTAB) [115], tannic acid [116], hydroxylamine hydrochloride [117], and polyvinylpyrrolidone (PVP) [118]. A reducing agent changes a metal ion in solution to a solid particle. For example, sodium borohydride (NaBH_4_) reduces Au^+3^ to solid Au in the Brust-Schiffrin method of gold nanoparticle synthesis [107]. Some common reducing agents used for gold nanoparticle synthesis include sodium citrate [106], NaBH_4_ [107], and hydroxylamine [69].

A common chemical reduction method is the Turkevich method. It was introduced in the early 1950s [106] and was a modification of a previous method to synthesize gold nanoparticles by Hauser and Lynn [119]. Although the Turkevich method was introduced over 60 years ago, it is one of the most commonly used methods today and elements of this method can be seen in many techniques to coat particles and surfaces with gold or silver. In this method, sodium citrate is added to a boiling aqueous solution of chloroauric acid (HAuCl_4_). Sodium citrate acts as the capping and reducing agent. The Lee-Meisel method [108] uses the same concept to form silver nanoparticles by reducing silver using sodium citrate. The Turkevich and Lee-Meisel methods produce irregularly shaped nanoparticles [120, 121] with diameters ranging from 10 to 150 nm [122] and 60 to 200 nm [120], respectively. It should be noted that several adaptations have been incorporated to these methods to improve control of nanoparticle size and shape [120, 123].

Nanoparticle size is most affected by the strength and concentration of the reducing agent. Stronger reducing agents produce smaller nanoparticles and weaker reducing agents producing larger particles [124]. Sodium citrate is a weak reducing agent, and as such, the Turkevich and Lee-Meisel methods produce relatively large nanoparticles. The Brust-Schiffrin method [107] uses sodium borohydride as a reducing agent, which is much stronger. As a result, this method creates well-dispersed gold nanoparticles ranging from 1 to 3 nm [107].

The size distribution of particles can be refined by controlling the nucleation and growth stages during chemical reduction. In the nucleation process, metal atoms combine to form clusters and finally crystal nuclei. In the growth step, the crystal nuclei or “seeds” grow in size to form nanoparticles. These steps can be separated for greater control of size and shape and the process is referred to as seed-mediated growth. In these methods, direct synthesis is used to produce the seeds. Consistent seeds are vital to control subsequent nanoparticle shape and size [115, 125, 126]. Several journal articles give further details of seeding methods [115, 120, 125–129] and will not be covered here.

Nanoparticle shape can be controlled by adding surfactants during synthesis which will cause a change in surface energy and control particle aggregation. The surfactant will stabilize specific crystal planes in the growing nanostructure, allowing controlled growth on that plane [130]. By carefully choosing a surfactant and particle material, multiple nanoparticle shapes have been created including nanorods [111, 131], nanocubes [132], nanostars [92, 133], nanotriangles [36, 134], nanoplates [135], nanowires [136], and nanoshells [137–139].

## 5. Tailoring Nanoparticles for Extrinsic SERS Applications

### 5.1. Coating Extrinsic Nanoparticles

A common approach for coating Raman reporter encoded gold nanoparticles is to bind a thiol PEG molecule to the gold nanoparticle surface by a gold thiolate bond. For citrate stabilized gold nanoparticles, the thiol group will easily replace the citrate molecule and will provide stabilization [140]. The surface charge of the particles can be controlled by varying the molecular weight of PEG. A simple one-step method to synthesize PEG coated gold nanoparticles has recently been developed [141]. Briefly, it involves heating an aqueous PEG and NaOH solution at 50°C. An aqueous solution of HAuCl_4_ is then added rapidly while slowly increasing the temperature to 80°C. This method avoids the two-step process of making the gold nanoparticles and then coating with PEG. It also avoids the use of additional chemicals as reduction agents.

The use of a silica shell for coating Raman reporter encoded nanoparticles provides increased stability even in the presence of multiple solvents [142]. To coat the Raman reporter labeled nanoparticles, aminosilanes, such as (3-aminopropyl)triethoxysilane (APTES) or (3-aminopropyl)trimethoxysilane (APTMS), are used to functionalize the particle surface. Tetraethyl orthosilicate (TEOS) or another activated cross-linking agent is used to form the silca shell. TEOS is highly sensitive to water and forms silica dioxide when added to the aminosilane activated gold nanoparticles [143]. Upon formation of the silica shell, these particles are extremely stable and have been used for* in vivo* SERS measurements without loss of the SERS response [16].

### 5.2. Addition of Antibodies or Other Affinity Ligands

An important factor in the design of extrinsic SERS nanoparticles' is the ability for targeting of specific recognition sites through antibody antigen interactions [15] or DNA hybridization [143]. To bind these ligands to the particle shell, multiple techniques have been used [138, 143–145]. A common approach is the use of a PEG N-hydroxysuccinimide (NHS) ester for covalent linkage to primary amines in the N terminus or lysine residues. For additional information see [146]. Ligands can be bound to silica coated particles using (3-glycidoxypropyl)methyldiethoxysilane (GPTMS). Li et al. [143] used GPTMS to provide a link between a silica surface and DNA. This binding is due to a highly reactive epoxide group associated with GPTMS. Another approach of surface functionalization involves the addition of a carboxyl group to the surface of the silica shell for further bioconjugation through EDC carbodiimide and NHS chemistry. In this approach, the nanoparticles are first suspended in dimethylformamide (DMF) and then 3-(triethoxysilylpropylcarbamoyl)butyric acid is added to the nanoparticles' silica surface. The resulting COOH functionalized silica particles can be used for bioconjugation of oligonucleotides or antibodies [147].

## 6. Future Applications

### 6.1. Hybrid Intrinsic/Extrinsic SERS

A potential application of SERS nanoparticles is the development of hybrid nanoparticles. Extrinsic methods generally use Raman reporters and antibodies for indirect sensing of the target molecule, while intrinsic methods directly enhance the target molecules' Raman spectrum. Hybrid applications combine elements of both intrinsic and extrinsic methods. This results in a nanoparticle which can target and enhance the Raman signature of a molecule providing structural information. An approach to develop hybrid SERS nanoparticles is to expose parts of the nanoparticle surface so that it can interact with the target molecule. Kneipp et al. [148] used unprotected nanoparticles with bound reporter molecules to investigate cellular uptake and endosome formation inside various different cells [148–150]. The presence of the reporter molecule enabled multiplexing and specific particle localization. Additionally, the unprotected particle surface allowed the detection of Raman bands related to intracellular components such as lipids, nucleotides, and proteins [149].

A critical factor in the design of hybrid nanoparticles is minimizing the distance between the particle and the target molecule. As mentioned previously, the target molecule must be within approximately 1–30 nm for SERS enhancement to occur. Several researchers have applied different techniques to minimize the distance between the nanoparticle and the target using hybrid SERS applications. For example, Hodges et al., used antibody conjugated gold nanoparticles to bind to specific cell surface regions for targeted SERS enhancement [151]. The large size of the antibody resulted in a large separation between the nanoparticle and the target molecule which limited the SERS enhancement. In order to increase the SERS enhancement, the distance between the metal surface and the target molecule was reduced by using the gold nanoparticles as nucleation centers for silver deposition. The deposited silver allowed for SERS enhancement of only the region around the antibody binding site [151]. Another technique to minimize the distance between the nanoparticle and the target molecule is through the use of antibody fragments. An antibody fragment is composed of the antigen binding site but does not contain the tail region. As a result the binding capacity is preserved but the total distance occupied by the fragment is less than that of a full antibody. Wang et al. demonstrated that nanoparticles with conjugated antibody fragments can significantly increase the SERS intensity while still maintaining targeting capabilities [152].

## 7. Summary

The optimization of SERS nanoparticles to achieve the highest enhancement factor is a complex process that incorporates multiple variables which must be precisely controlled by synthesis methods. Nanoparticles for intrinsic SERS applications can be synthesized to control composition, size, shape, and configuration which will affect the LSPR peak location and SERS EF. Smaller particles result in a shorter LSPR wavelength in accordance with the Mie theory. Size can be set during chemical synthesis by proper control of nucleation and growth steps as well as the strength of the reducing agent. Stronger reducing agents will result in smaller particles. Shape will also influence SERS enhancement due to electromagnetic enhancement at edges or sharp corners. Shape can be controlled during synthesis by the addition of surfactants. The proper choice of Raman reporters and protective coatings will determine the enhancement associated with extrinsic SERS. An appropriate Raman reporter will have a strong binding affinity to the nanoparticle surface and allow for resonance Raman enhancement. Care must be taken in the synthesis of the protective layer to prevent loss of the SERS signal. Controlled synthesis of intrinsic and extrinsic SERS nanoparticles can be tuned for specific systems and applications providing optimal SERS enhancement.

## Figures and Tables

**Table 1:** A list of molecules used for SERRS enhancement for excitation wavelengths (*λ*_0_) from 514–785** **nm and their associated absorption maximums (*λ*_max⁡_).

Raman reporter	*λ*_max⁡_ (nm)	*λ*_0_ (nm)	Reference
Carboxyfluorescein (FAM)	494	514.5	[85]
Rhodamine 6 G (R6G)	524	514.5, 532	[85–88]
TRITC-DHPE	540	514.5	[89]
Carboxy-X-rhodamine (ROX)	585	514.5, 632.8	[85]
BIODIPY TR-X	588	632.8	[85]
Crystal violet (CV)	590	514.5, 647.1	[86, 90]
Malachite green (MG)	618	632.8	[91]
Malachite green isothiocyanate (MGITC)	625	632.8	[91]
Methylene blue	570–760^∤^	785	[92, 93]
Cy5.5	683	632.8	[85]
3,3′-diethylthiatricarbocyanine iodide (DTTC)	765	785	[94]

^∤^Methylene blue (MB) has various different maximum absorption points due to the formation of molecular aggregates and protonation states. The formation of dimer and trimer aggregates and MB protonation causes a shift in the maximum absorption. As a result, the maximum absorption is directly related to the state of the MB molecule during measurement [93].
